# Correlation between Handgrip Strength and Rapid Shallow Breathing Index for Assessment of Weaning from Mechanical Ventilation

**DOI:** 10.1155/2021/4637528

**Published:** 2021-11-30

**Authors:** Narongkorn Saiphoklang, Thanapon Keawon

**Affiliations:** Division of Pulmonary and Critical Care Medicine, Department of Internal Medicine, Faculty of Medicine, Thammasat University, Bangkok, Thailand

## Abstract

**Background:**

Assessment of weaning from mechanical ventilation (MV) is an important process. Rapid shallow breathing index (RSBI) is a standard tool to evaluate a patient's readiness before the spontaneous breathing trial (SBT). Handgrip strength (HGS) is an alternative method for assessment of respiratory muscle strength. Relationship between HGS and RSBI has not been explored. This study aimed to determine the correlation between HGS and RSBI to predict successful extubation in mechanically ventilated patients.

**Methods:**

A prospective study was conducted in screened 120 patients requiring MV with tracheal intubation >48 h. HGS was performed at 48 h after intubation, 10 min before and 30 min after SBT, and 1 h after extubation. RSBI was performed at 10 min before SBT.

**Results:**

A total of 93 patients (58% men) were included in the final analysis. Mean age was 71.6 ± 15.2 years. Patients admitted in general medical wards were 84.9%. APACHE II score was 13.5 ± 4.7. Most patients were intubated from pneumonia (39.8%). Weaning failure was 6.5%. The main result shows that HGS was negatively correlated with RSBI (regression coefficient −0.571, *P* < 0.001). The equation for predicting RSBI, derived from the linear regression model, was predicted RSBI (breaths/min/L) = 39.285 + (age *∗* 0.138)–(HGS *∗* 0.571).

**Conclusions:**

HGS had significantly negative correlation with RSBI for assessment of weaning from MV. A prospective study of the HGS cutoff value is needed to investigate the difference between patients who succeeded and those who failed extubation. This trial is registered with TCTR20180323004.

## 1. Introduction

Most patients require mechanical ventilation (MV) with intubation due to respiratory failure. Assessment of their readiness for ventilator weaning is a crucial approach to reduce complications and mortality [1].

The use of rapid shallow breathing index (RSBI) has increased widely due to its simple method and avoidance of calculation of complex pulmonary mechanics [2]. It is the most accurate predictor of failure in weaning patients from MV [3] and is the most frequently studied and seems to be an important measurement tool in deciding whether to wean or extubate a patient [4]. However, the results of several studies revealed that weaning and extubation should be guided by several parameters and not only by respiratory ones [4]. Besides RSBI, several parameters can predict weaning success, e.g., handgrip strength (HGS), heart rate variability, sleep quality, diaphragmatic dysfunction, and biomarkers [5]. HGS may be a useful tool for the weaning assessment from prolonged MV at a long-term acute-care hospital, in addition to the maximum inspiratory pressure (PImax) [6]. During 6-month follow-up, HGS increased 6.2 times compared to HGS at recruitment. During the same period, PImax also increased but to a lesser degree [6]. Moreover, hand grasping and tongue protrusion used as simple motor tasks were predictors of failed extubation in critically ill neurologic patients if they could not follow simple motor commands [7]. HGS can predict difficult or prolonged weaning in mechanically ventilated patients, supported by a previous study that showed different HGS between weaning groups: 20 kg, 12 kg, and 6 kg for simple, difficult, and prolonged weaning, respectively [8]. Low strength was correlated with significantly increased reintubation rate [9]. In the previous study, patients in the reintubation group had significantly lower HGS at 10 min and 30 min after starting spontaneous breathing trial (SBT) (7.6 ± 4.8 kg vs. 13.4 ± 6.5 kg and 8 ± 5.1 kg vs. 13.2 ± 5.7 kg) compared to the non-reintubation group [9]. Furthermore, intensive care unit-acquired paresis was associated with lower HGS, poor hospital outcomes, and in-hospital mortality [10]. HGS was also correlated with respiratory muscle strength in the healthy elderly, as well as the maximal inspiratory pressure and maximal expiratory pressure [11].

We hypothesized that HGS indicates respiratory muscle strength and may be correlated with RSBI, an established predictor of successful extubation. This study aimed to evaluate the correlation between HGS and RSBI for weaning from MV.

## 2. Materials and Methods

### 2.1. Study Design and Subjects

A prospective study was conducted between January 2018 and January 2020 in general medical wards and a medical intensive care unit (ICU) at Thammasat University Hospital, Thailand. Patients requiring MV with tracheal tube intubation for at least 48 h and aged 18 years or older were recruited. All recruits were able to cooperate fully, able to do the HGS test, and were expected to pass the SBT upon extubation from MV. Exclusion criteria were death before weaning from MV, less than 48 h of MV, transfer to other hospitals, self-extubation, accidental extubation, reintubation before enrollment, undergoing tracheostomy, inability to perform the HGS test, and treatment with vasopressor/inotropic drugs. The 34 patients successfully recruited for this study were also among the patients who participated at the same time in our previously published study [9].

The weaning process was under the supervision of the attending physicians. However, all members of the team who participated in the weaning process underwent prestudy training on weaning according to the standards of the European and American Respiratory/Intensive Care Societies [1]. Weaning was attempted as early as possible during the patients' illnesses with a two-step approach in which readiness for weaning was assessed daily according to the standard criteria of the European and American Respiratory/Intensive Care Societies [1]. Patients who fulfilled these criteria underwent SBT. The duration of SBT was 30–120 min and consisted of either breathing with a T-piece or a weaning trial undergoing 5–8 cm H_2_O pressure support with 5 cm H_2_O-positive end-expiratory pressure. When patients successfully passed the SBT, the physician in charge, in collaboration with the attending medical staff initiated the weaning process. If a patient failed the initial SBT, MV was reinstituted, and the physician reviewed the possible reversible causes of the weaning failure, e.g., bronchospasm, secretion obstruction, pulmonary edema, congestive heart failure, psychoneurological factors, electrolyte imbalances, malnutrition, and anemia. SBT was repeated the following day if the patient then appeared ready for weaning. A patient was rated as successfully weaned when he or she was extubated and breathing spontaneously without any invasive or noninvasive ventilatory support for ≥48 h. Concordantly, weaning failure was defined as either the failure of SBT or the need for reintubation within 48 h following extubation [1].

Participants were classified into 3 weaning groups: (1) simple weaning: successful weaning and extubation on the first attempt without difficulty, (2) difficult weaning: failure of initial weaning and the need for up to three SBTs for as many as 7 days from the first SBT to achieve successful weaning, and (3) prolonged weaning: failure of 3 or more weaning attempts or the need for longer than 7 days of weaning after the first SBT. [1].

Demographics and baseline characteristics including acute physiology and chronic health evaluation (APACHE) II scores were collected for all participants. During SBT, data on MV weaning were collected including vital signs, RSBI, type of SBT, time of SBT success, and time of extubation. HGS was tested at 48 h after intubation and 10 min and 30 min after SBT. In addition, this measurement was performed at 1 h after extubation. RSBI and HGS test were both performed at 10 min before SBT. The physician in charge did not know HGS results.

Ethic approval was obtained from the Human Ethics Committee of Thammasat University (IRB No. MTU-EC-IM-0-198/60), in compliance with Declaration of Helsinki, The Belmont Report, CIOMS Guidelines, and The International Practice (ICH-GCP). All methods were performed in accordance with these guidelines and regulations. All participants provided written informed consent.

### 2.2. Outcomes and Measurements

Primary outcome was the association between HGS and RSBI, adjusted for age, sex, and APACHE II score. Secondary outcomes were the difference in HGS between weaning success and weaning failure groups and the differences in HGS between simple, difficult, or prolonged weaning groups. Maximal grip strength from 3 efforts for each hand was recorded using a specialized dynamometer (Jamar, Asimow Engineering Co., Santa Monica, CA, USA), The measurements were made at rest with the hand unsupported, with the elbow at 90° flexion, and with the wrist in neutral position. RSBI was calculated by patient's respiratory rate divided by tidal volume (breaths/min/L), which were obtained while the patient was undergoing MV with 5 cm H_2_O positive end-expiratory pressure and no pressure support.

### 2.3. Statistical Analysis

The sample size was calculated on the basis of our hypothesis expecting the correlation coefficient between HGS and RSBI of 0.5. Thus, 38 patients would provide a power of 90% and a two-sided alpha level of 5%. Categorical data were shown as number (%). Continuous data were shown as mean ± standard deviation. Student' *t*-test was used to compare continuous variables between two groups. ANOVA was used for comparison of 3 weaning groups. To determine the set of variables associated with RSBI, we used the linear regression model with the RSBI set as dependent variable. All independent variables (age, sex, HGS, and APACHE II score) were entered into the regression model simultaneously. We report the regression coefficients, their 95% confidence interval, and corresponding *p* values. Variables with *p* value <0.05 were considered statistically associated with RSBI. Using the regression coefficients and the intercept, predicted RSBI for a patient could be calculated from the following equation, where *V* represents the covariate, *β* represents the regression coefficient, and *i* represents the number of variables:

(1) $$\begin{array}{c} \text{Predicted RSBI}=\text{intercept}+V1\beta 1+V2\beta 2+Vi\beta i. \end{array}$$

Statistical analyses were performed using SPSS version 20.0 software (IBM Corp., Armonk, NY, USA).

## 3. Results

### 3.1. Patients

**Figure 1:**
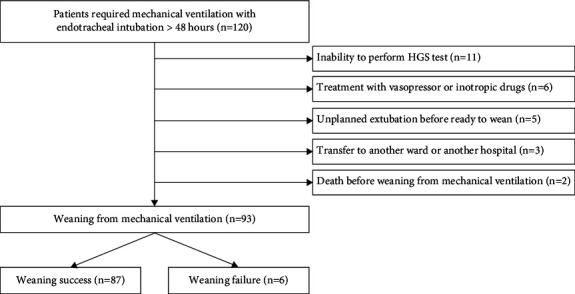
Study flowchart indicating inclusion and exclusion population.

One hundred twenty mechanically ventilated patients were recruited, and 93 of these were included in the final analysis (Figure 1). There were 54 (58.1%) males. Mean age was 71.6 ± 15.2 years. Most patients were admitted in general medical wards (84.9%, 79 of 93 patients). APACHE II score was 13.5 ± 4.7. Most patients were intubated from pneumonia (39.8%, 37 of 93 patients). The most common type of SBT was pressure support ventilation (74.2%, 69 of 93 patients) (Table 1). The highest HGS was at 1 h after extubation (16.3 ± 6.5 kg). RSBI at 10 min before SBT was 40.2 ± 9.2 breaths/min/L. Weaning failure was 6.5% (6 of 93 patients) (Table 2). The causes for extubation failure were pneumonia (33.3%, 2 of 6 patients), pulmonary edema (16.7%, 1 of 6 patients), marked airway secretions (16.7%, 1 of 6 patients), bronchospasm (16.7%, 1 of 6 patients), and hypoalbuminemia (16.7%, 1 of 6 patients). Incidence of simple, difficult, and prolonged weaning was 77.4%, 20.4%, and 2.2% (72, 19, and 2 of 93 patients), respectively.

### 3.2. Primary Outcome

**Figure 2:**
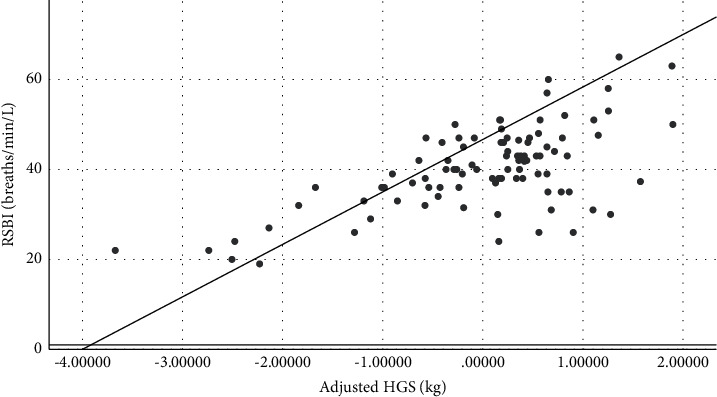
Linear regression analysis showing the correlation between rapid shallow breathing index (RSBI) and handgrip strength (HGS) at 10 min before spontaneous breathing trial (SBT) adjusted by age, gender, and acute physiology and chronic health evaluation II score. Predicted RSBI = 39.285 + (age *∗* 0.138)–(HGS *∗* 0.571), regression coefficient: −0.571, *P* < 0.001.

The main result shows that HGS was negatively correlated with RSBI. The equation for predicting RSBI, derived from the linear regression model, was predicted RSBI (breaths/min/L) = 39.285 + (age *∗* 0.138)–(HGS *∗* 0.571), with regression coefficient −0.571, *P* < 0.001 (Figure 2 and Table 3).

### 3.3. Secondary Outcome

HGS was significantly higher in patients in the weaning success group than in the weaning failure group over time. However, RSBI did not significantly differ between the two groups (Table 2).

At 10 min before SBT in simple, difficult, and prolonged weaning groups, maximum HGS was 16.3 ± 7.2, 13.7 ± 4.7, and 15.1 ± 0.4 kg, respectively (*P*=0.202), and RSBI was 39.3 ± 9.7, 43.6 ± 6.7, and 39.0 ± 9.2 breaths/min/L, respectively (*P*=0.318).

For predicting successful extubation, the best HGS cutoff value at 10 min before SBT was 12.7 kg with area under the receiver operating characteristic (ROC) curve of 0.842 (95% CI: 0.67–1.00, *P*=0.005). We observed a 75.9% sensitivity and 83.3% specificity for the 12.7 kg HGS cutoff. The best RSBI cutoff value at 10 min before SBT was 43.5 breaths/min/L, with area under the ROC curve of 0.459 (95% CI: 0.16–0.75, *P*=0.737), 33.3% sensitivity, and 66.6% specificity.

## 4. Discussion

This study is the first prospective study that revealed the correlation between HGS and RSBI to predict successful extubation in mechanically ventilated patients. It differs from our previously published study [9] which showed that low HGS was correlated with the significantly increased reintubation rate in mechanically ventilated patients. The 34 patients recruited for this current study also participated in our previously published study at the same time. Majority of our patients (85%) were admitted in general medical wards due to the limitation of ICU beds in our hospital. However, there were sufficient devices for respiratory and noninvasive hemodynamic monitoring in general medical wards. Our research with patients in a mixed population from general medical wards and a medical ICU revealed that HGS had a significantly negative correlation with RSBI for assessment of the weaning process. Furthermore, this study derived the equation predicting RSBI by using the HGS factor to determine the weaning outcome. Several studies had explored HGS in critically ill patients receiving MV, but these studies did not investigate the relationship between RSBI and HGS [7, 10, 12].

A study by Yang and Tobin determined that RSBI is an accurate index to predict weaning failure (a threshold of 105 breaths/min/L with 78% positive predictive value and 95% negative predictive value) which is more accurate than CROP index (dynamic compliance, respiratory rate, oxygenation, and maximum inspiratory pressure index), minute ventilation, or maximal inspiratory pressure [3]. However, some patients will require reintubation and institution of MV despite meeting established weaning criteria, while some patients not meeting criteria can be successfully liberated from the ventilator. Our RSBI was smaller, 70 breaths/min/L, than in Yang and Tobin' study [3], which may have resulted from the fact that most of our patients were elderly (mean aged 71 years old) and some patients had COPD; thus, these patients might have had air trapping and high lung volume leading to lower RSBI. Moreover, RSBI did not differ between those who were successful in extubation and those that failed in our study. We speculated that the RSBI values could have been confounded by factors other than respiratory muscle strength (e.g., increased respiratory rates from pneumonia and low tidal volume in pulmonary edema), leading to misleading results. Meanwhile, HGS is directly correlated with respiratory muscle strength with less interference from other factors.

Mohamed-Hussein AAR et al., in a study comparing HGS at weaning to intensive care outcomes in 34 patients with chronic obstructive pulmonary disease (COPD), found that HGS may be a good predictor for extubation outcomes as well as MV duration and ICU mortality [12].

A previous study of Cottereau G et al. demonstrated that there were statistically significant differences in HGS between simple, difficult, and prolonged weaning groups (20 kg, 12 kg, and 6 kg, respectively, *P*=0.008), but no association was found between HGS and extubation failure [8]. A previous study by Saiphoklang and Auttajaroon showed that there were significant differences in mortality rates between 3 weaning groups, but HGS was not investigated [13]. In contrast, our study shows there was no difference in HGS between the 3 weaning groups, but there was a significant difference in HGS between weaning success and weaning failure groups. The first reason for the findings may be because the previous study [8] was conducted only in ICU which needed more intensive and close monitoring after weaning or extubation such as airway care and pulmonary rehabilitation, unlike this study which included over 50% of patients from general medical wards. Another reason is that our patients in this study were recruited from a more aging population (mean age of 71 years), and the main indications for MV were respiratory conditions such as pneumonia, airway protection, and COPD exacerbation, which may have had direct effects on weaning processes.

This study has a few limitations. The first is that this study cannot demonstrate the cut point of HGS for extubation failure. The second limitation is limited postextubation monitoring in general wards as discussed above. The last is that this study included only patients from medical wards and thus may not apply to surgical patients. A future study may find the cut point of HGS of extubation success in order to implement it to a ventilator liberation protocol.

## 5. Conclusions

HGS had significantly negative correlation with RSBI for assessment of the weaning process in mechanically ventilated patients. A prospective study of the HGS cutoff value is needed to investigate the difference between patients who succeeded and those who failed extubation.

## Figures and Tables

**Table 1:** Baseline characteristics.

Characteristics	*N* = 93
Age, years	71.6 ± 15.2

Male	54 (58.1)

Body mass index, kg/m^2^	23.5 ± 4.6

Dominant right hand	87 (93.5)

Ward	
General medical wards	79 (84.9)
Medical intensive care unit	14 (16.1)

Underlying disease	
Diabetes	70 (75.3)
Hypertension	13 (14)
COPD	15 (16.1)
Malignancy	13 (14)
Chronic kidney disease	8 (8.6)
Chronic heart failure	7 (7.5)
APACHE II score, points	13.5 ± 4.7

Indication for intubation with mechanical ventilation	
Pneumonia	37 (39.8)
Airway protection	12 (12.9)
AECOPD	11 (11.8)
Congestive heart failure	9 (9.7)
Volume overload	6 (6.5)
Septic shock	2 (2.2)
Atrial fibrillation	1 (1.1)
Others	7 (7.5)

Mode of ventilator weaning	
Pressure support ventilation	69 (74.2)
T-piece	24 (25.8)

Data are presented as n (%) or mean ± SD. AECOPD, acute exacerbation of COPD; APACHE II, acute physiology and chronic health evaluation II; COPD, chronic obstructive pulmonary disease.

**Table 2:** Comparison of handgrip strength and rapid shallow breathing index between weaning success and weaning failure.

Variable	Total, *n* = 93	Weaning success, *n* = 87	Weaning failure, *n* = 6	*P* value
HGS at 48 h after intubation, kg	15.6 ± 7.0	16.2 ± 7.3	6.8 ± 4.7	0.002
HGS at 10 min before SBT, kg	15.8 ± 6.7	16.3 ± 6.5	8.2 ± 5.3	0.004
HGS at 30 min before SBT, kg	15.9 ± 6.6	16.4 ± 6.3	8.6 ± 5.6	0.004
HGS 1 h after extubation, kg	16.3 ± 6.5	16.8 ± 6.3	8.6 ± 5.5	0.002
RSBI at 10 min before SBT, breaths/min/L	40.2 ± 9.2	40.0 ± 8.9	42.2 ± 14.4	0.590

Data are presented as mean ± SD. HGS, handgrip strength; RSBI, rapid shallow breathing index; SBT, spontaneous breathing trial.

**Table 3:** Linear regression analysis for rapid shallow breathing index and handgrip strength, age, gender, and APACHE II score.

Variables	Regression coefficients	95% CI of coefficients	*P* value
Intercept	39.285	27.491, 51.079	<0.001
HGS at 10 min before SBT	−0.571	−0.834, −0.309	<0.001
Age	0.138	0.033, 0.242	0.010
Male	−3.119	−6.466, 0.229	0.067
APACHE II score	0.138	−0.215, 0.491	0.439

HGS, handgrip strength; SBT, spontaneous breathing trial.

## Data Availability

The data used to support the results of this study are included within the article.
